# A Glimmer of Hope: The Impact of the Recovery College Bern on Personal Recovery, Well‐Being and Self‐Stigmatisation—A Mixed Methods Study

**DOI:** 10.1111/inm.13482

**Published:** 2024-12-04

**Authors:** Nora Ambord, Christian Burr, Gianfranco Zuaboni

**Affiliations:** ^1^ Department of Health Professions Bern University of Applied Sciences Bern Switzerland; ^2^ Recovery College Bern Switzerland; ^3^ University of Lucerne Department of Health Sciences and Medicine Lucerne Switzerland; ^4^ University Clinic of Psychiatry and Psychotherapy Bern University Hospital for Mental Health Bern Switzerland; ^5^ Psychiatric and Psychotherapy Hospital, Sanatorium Kilchberg AG Kilchberg Switzerland

**Keywords:** mental health recovery [MeSH], program evaluation [MeSH], psychological well‐being [MeSH], recovery college, self‐stigmatisation

## Abstract

Recovery Colleges are mental health education centres co‐produced by experts with lived experience with mental health problems and mental health professionals. The aim of the study was to evaluate the impact of a Recovery College in Switzerland on its students' mental health measured through personal recovery, well‐being and self‐stigmatisation in a mixed methods approach following the MMARS guideline. Three standardised questionnaires ‘Questionnaire about the Process of Recovery’, ‘WHO‐5 Well‐Being Index’ and ‘Self‐Stigma of Mental Illness Scale Short Form’ were completed by 92 participants as part of a pre‐post‐evaluation while two focus groups (*n* = 10) provided further explanations regarding impacts on the three topics. Statistical analyses include paired sample *t*‐test or Wilcoxon signed rank tests for pre‐post‐test comparisons as well as Cohen's d to determine effect sizes. For all three questionnaires, significant improvement was shown in the desired direction with low to medium effect sizes. A higher number of courses attended did not result in higher scores in the outcome measurements. The qualitative analysis confirmed these results by providing insights of specific aspects of these positive impacts. These include increased social inclusion, improvement in attitudes towards one's life and identity, increased engagement in hobbies and healthy behaviours, positive impacts on well‐being and decreased self‐stigmatisation. The findings indicate that Recovery Colleges should be made available continuously and further developed also in other regions of Switzerland. Similar projects require continuous evaluation in early development to ensure effectiveness and improve quality.

## 1 Introduction

In the past decade, personal recovery has emerged as a relevant issue of psychiatric service provision supported by the World Health Organisation through their strong recommendation in the action plan 2013–2030 for mental health (WHO 2021) as well as in the quality rights programme (WHO 2019), advocating for its inclusion in global health planning. Personal recovery is defined as a person's unique process of personal change, leading to a satisfying, hopeful, and contributing life even with the limitations caused by their illness (Anthony 1993). This differs from the traditional definition of clinical recovery in mental health services which mainly focuses on symptom reduction and increased functioning (Slade, Amering, and Oades 2008). A literature review (Leamy et al. 2011) summarised the further developments in research of personal recovery into a conceptual framework containing five core processes: connectedness, hope, identity, meaning and empowerment (CHIME).

Including the central aspects of personal recovery, Recovery Colleges (RC) can be seen as a reinforcement of the development to increased recovery orientation in mental health services (Perkins and Repper 2017). RCs are education centres offering courses covering various aspects of psychological health with a strong focus on helping to manage and navigate daily living (Lin et al. 2023). The first RC opened in 2009 in the UK and since then at least 22 countries all over the world have followed (Hayes et al. 2023; Thériault et al. 2020). Using an educational approach rather than clinical or therapeutic methods, RCs are co‐designed and co‐facilitated by mental health professionals and experts by lived experience supporting people with mental health issues, their families and careers, staff and everyone interested (Perkins et al. 2012). People with lived experience are involved in all parts of the organisation, such as the development of the curriculum, the course coordination as well as the course delivery (Gill 2014; Crowther et al. 2019). Co‐produced programmes help facilitate a culture of equity and inclusion and it has been shown that they may lead to positive shifts in knowledge, attitudes and behavioural intentions of people with mental health issues as well as healthcare staff (Lewis et al. 2017; Whitelaw et al. 2022). On a societal level, RCs aim to increase community inclusion and participation as well as to reduce discrimination and stigmatisation which leads to decreased self‐stigmatisation, a very common phenomenon among people with mental illness (Crowther et al. 2019).

The Recovery College Bern (RCB) was founded in collaboration with a psychiatric University Hospital in Switzerland in 2019. It offers a bi‐semester programme with over 60 courses on several topics that can be broadly categorised as follows: Strengthening mental health; understanding and dealing with the impact of mental health problems in daily life as well as supporting personal development. Students in the RCB may choose courses according to their preferences and goals varying from short, 1‐h introductory courses to longer courses lasting several hours with up to 7 sessions. Since its launch, nearly 1000 people have participated in the RCB and in addition to the project team over 50 trainers are currently working on mandatory bases to develop and deliver their courses in co‐production. The RCB is the first Recovery College in the German‐speaking part of Switzerland.

As RCs have only emerged quite recently, their evidence base is still limited (Ali, Benkwitz, and Mcdonald 2022). Nevertheless, evaluations conducted over the last decade have shown several positive impacts of RCs, such as increased well‐being and decreased self‐stigmatisation (Nurser, Hunt, and Bartlett 2017) as well as a decrease in mental health services use (Bourne, Meddings, and Whittington 2018). In their practical implications, Meddings et al. (2015) recommend using both quantitative and qualitative designs to attain a comprehensive evaluation of RCs. Mixed methods evaluations of RCs have shown to give a better understanding of results through the complementary qualitative data (Thériault et al. 2020).

This study's primary objective is to evaluate the impact of the RCB on its students with mental health problems regarding personal recovery, well‐being and self‐stigmatisation. Additionally, it seeks to evaluate whether a higher number of attended courses strengthens improvements in the measured outcomes.

## 2 Methods

### 2.1 Design

A mixed methods pre‐post evaluation design was chosen with the goal to gain more insights and to receive explanations of the quantitative results through qualitative data in a complementary approach. The mixed methods design has been recommended to bring more value to a study as a whole (Small 2011). The present article was written in accordance with the Mixed Methods Article Reporting Standards (MMARS) following EQUATOR recommendations (Levitt et al. 2018). The design includes:

1. A quantitative part: A pre‐post intervention group design with standardised questionnaires measuring personal recovery, well‐being and self‐stigmatisation of participants; the baseline being completed before the first attended course and follow‐up questionnaires being completed at the end of each course.
2. A qualitative part: Two focus groups involving participants who have completed courses further discussing the topics of personal recovery, well‐being, self‐stigmatisation and the conduct of the evaluation.

### 2.2 Participants

For the quantitative part, all students were included who agreed to complete the routine evaluation questionnaires and attended at least 80% of a RCB course with 3 or more sessions. For this study, only course participants with self‐reported mental health problems were included. As the minimum age to attend the RCB is 16 years, participants from this age were included without any other restriction. For the qualitative evaluation, people who were part of the quantitative evaluation were asked to participate in a focus group.

### 2.3 Ethics

The responsible ethics committee concluded that ethics approval was not required for this study (Req‐2021‐01293) as it is not subject to the Human Research Act in Switzerland. The data were anonymised with only the main author having access to the key to unlock anonymisation. Before collecting data, all participants received oral and written study information, and written consent was obtained. We did not register the study because it was a part of an ongoing evaluation of the RCB.

### 2.4 Quantitative Data Collection

The quantitative data were collected between April 2021 and December 2022. To be included, participants had to complete at least two questionnaires at different time points, namely at baseline and follow‐up. As all participants were always given a post‐questionnaire at the end of each course, some may have completed more than two questionnaires in total but for this analysis, only the first and the last one were used. A participant code was allocated to each person to ensure the matching of the pre‐ and post‐questionnaires while maintaining confidentiality. Additionally, it was verified how many courses each of the participants had completed between their pre‐ and post‐questionnaires.

### 2.5 Quantitative Measures

The questionnaires used for the pre‐and post‐data collection were identical, except that at baseline gender and age were asked additionally. The following standardised self‐rated measures were used:

### 2.6 Questionnaire About the Process of Recovery (QPR)

The original Questionnaire about the Process of Recovery (QPR) consists of 22 self‐reported items measuring all five areas of the CHIME framework of personal recovery (Lanyon and Petrakis 2023). The QPR was developed in collaboration of service users and clinicians. In the underlying study, the German version (Elhilali et al. 2023) of the shortened 15 items one‐factor model version was used (Law et al. 2014). The QPR German Version shows excellent reliability values and model fit test (Elhilali et al. 2023). The Cronbach's alpha for internal consistency was reported with a value of 0.91 and the corrected item correlation with values between 0.49 and 0.73. RMSEA, CFI and TLI indices shows acceptable fit of the used model for analysis. The items are rated on a five‐point Likert scale ranging from 0 to 4 and ‘strongly disagree’ to ‘strongly agree’ respectively. A higher score indicates a higher level of recovery. A mean score difference of at least 5 points (8.3%) is suggested for clinical relevance within subjects (Dehmahdi et al. 2021).

### 2.7 WHO‐Five Well‐Being Index (WHO‐5)

The WHO‐5 consists of five positively phrased items measuring overall psychological well‐being. It is a short 5‐item rating scale version that was derived from a 28‐item version (Topp et al. 2015). The respondents are asked to rate how statements best describe their experience over the last 14 days on a six‐point Likert scale ranging from the scores 0 (‘at no time’) to 5 (‘all the time’) such as: I have felt cheerful and in good spirits; I woke up feeling fresh and rested; etc. Higher scores on the measure are indicative of a higher level of psychological well‐being. Topp et al. (2015) suggest a 10% mean score difference to reach clinical relevance in this questionnaire. For the underlying study, a validated German translation of the questionnaire was used for which statistical analysis showed excellent psychometric characteristics, including high reliability with an internal consistency of 0.92 (Brähler et al. 2007). The model demonstrated a good fit based on the RMSEA, CFI and TLI indices.

### 2.8 Self‐Stigma of Mental Illness Scale Short Form (SSMIS‐SF)

The Self‐Stigma of Mental Illness Scale is a 40‐item self‐report measure on attitudes related to self‐stigmatisation. For this study, the subscale on ‘application of self‐stigma’ of the shortened version of the Self‐Stigma of Mental Illness Scale (SSMIS‐SF) was used. In a previous psychometric evaluation from subsamples of three studies, this subscale demonstrated adequate alpha values for internal consistency with 0.74 and 0.69 in two of the three subsamples even if one subsample showed very low values with an alpha of 0.22 (Corrigan et al. 2012). This subscale consists of 5 items including statements all starting with the phrase ‘Because I have a mental illness I’. which for example ends in ‘am dangerous’. The respondents are asked to rate the statements on a nine‐point Likert scale ranging from ‘strongly disagree’ (1) to ‘strongly agree’ (9). A lower score indicates a lower level of self‐stigmatisation. There is no available recommendation for a minimum mean score difference necessary to achieve clinical relevance for the SSMIS‐SF. Although used in other studies (Hamann, Bühner, and Rüsch 2017), the German version of the SSMIS‐SF has not yet been validated.

### 2.9 Quantitative Analysis

For the statistical data analysis, IBM SPSS Statistics (version 14) was used. Descriptive analysis was conducted for the characteristics of the participants as well as the sum scores of the standardised measurements. Metric data were described in total mean scores and standard deviations, categorical data in absolute and relative frequencies. The power calculation of this study was done by g‐power (Faul et al. 2007) which suggested we would need 88 participants to achieve 90% power using a two‐tailed test assuming an effect size of 0.35 and an alpha error of 0.05. A Kolmogorov–Smirnov and Shapiro–Wilk test were used to determine normal distribution. A paired sample *t*‐test or Wilcoxon signed rank tests respectively was used to analyse within‐subject statistical differences between the pre‐ and the post‐sum scores of the questionnaires. Cohen's d was calculated to determine the effect size, whereas *r =* 0.2, 0.5 and 0.8 are considered small, medium and large, respectively (Cohen 1992). We further analysed the proportion of individuals in our sample who achieved the suggested percentage mean score difference necessary to attain clinical significance. For pragmatic reasons, we determined the threshold for clinical relevance in this calculation at a 10% mean score shift in the desired direction for all three questionnaires.

Furthermore, differences were tested between people who attended only one compared to two or more courses. For this, a new, dichotomous dummy variable was generated that differentiated between participants that attended only one course and participants that attended two or more courses. This distinction was chosen because most participants either visited one or two courses, resulting in two groups similar in size. Subsequently, the analysis of repeated measures ‘ANCOVA’ was performed to test for statistical differences between the sum scores of the two groups including the number of courses attended as a co‐variable (Seltman 2018). A Levene's test was conducted to test if the sum scores of the two time points had equal variance, which is described as a precondition for a repeated measure ANCOVA (Maxwell, Delaney, and Kelley 2017). If the Levene's test showed heterogeneity, no alternative analysis was conducted.

### 2.10 Qualitative Data Collection

Two focus groups were conducted in October of 2022. An interview guide was developed with questions aiming to get more in‐depth feedback on the impact of the RCB on the three topics of personal recovery, well‐being, and self‐stigmatisation and additional questions on the conduction of the evaluation. The focus groups took place on the premises of the RCB, with each session lasting approximately 1 h. Participants got a voucher for one free course in the RCB in return for participating.

### 2.11 Qualitative Analysis

The audio data of the focus groups were transcribed verbatim from Swiss German dialect to written German and were anonymised. Text passages were coded and categorised based on predetermined themes derived from the topics of the standardised questionnaires. Thematic analysis was then conducted deductively, following the structure outlined by Braun and Clarke (2006).

## 3 Results

Both the pre‐ and post‐questionnaire were completed by 92 students of the RCB and were therefore included in the quantitative data analysis. In the two focus groups, a total of 10 students (five each) participated. In both samples, about two‐thirds of the participants were female and about one‐third were male. The mean age in the qualitative sample was nearly 8 years higher than in the quantitative. In both samples, about half of the participants attended only one RCB course, while the other half had participated in two or more courses Tables 1, 2, 3.

**TABLE 1:** Quantitative study population (*n* = 92).

Variable	*n* (%)
Gender	
Female	59 (64.1)
Male	31 (33.7)
Others	2 (2.2)
Age	
Mean (SD)	42.9 (12.2)
Range	17–69
Number of completed courses	
1	54 (58.7)
2	21 (22.8)
3	8 (8.7)
4	6 (6.5)
> 5	3 (3.3)

**TABLE 2:** Qualitative study population (*n* = 10).

Variable	*n* (%)
Gender	
Female	7 (70)
Male	3 (30)
Others	0 (0)
Age	
Mean (SD)	50.6 (13.5)
Range	29–66
Number of completed courses	
1	5 (50)
2	1 (10)
3	3 (30)
4	1 (10)
> 5	0 (0)

**TABLE 3:** Descriptive statistics and *t*‐test/Wilcoxon signed rank test scores.

Variable	*n*	Min/Max	Mean pre (SD)	Mean post (SD)	*p*	*r*	Mean diff (%)	*n* (%) ≥ 10% mean diff. ****
QPR	92	0/60	40 (8.8)	42.5 (8)	< 0.001***	−0.395	2.5 (4.2)	25 (27.2)
WHO‐5	89	0/25	12.4 (5.1)	13.7 (5.1)	0.007**	−0.268	1.3 (4.8)	39 (42.4)
SSMIS‐SF	90	5/45	13.7 (7.2)	11.9 (7.3)	< 0.001***	0.269	−1.8 (4.4)	26 (28.3)

*Note:* *p < 0.05, **p < 0.01, ***p < 0.001 ****number of participants who reached 10 and more % mean difference of the total score of the corresponding scale (QPR: ≥ 6; WHO: ≥ 2; SSMIS‐SF: ≤ −4).

### 3.1 Personal Recovery

The sum score of the QPR increased from 40 to 42.5 (difference of 4.2%). The Wilcoxon analysis showed a significant improvement (*z* = 3.816, *p* < 0.001, *n* = 92) for these scores with a low to medium effect size (*r* = −0.395). Levene's test showed homogeneity for the variances (*p* = 0.668). After adjusting for the number of courses attended, the sum scores of the QPR did not differ statistically between participants that attended one course compared to participants that attended two or more courses (*F*(1, 90) = 0.208, *p* = 0.649, partial *η* ^2^ = 0.02).

During the focus groups, participants mentioned several improvements regarding the five personal recovery‐related themes: connectedness, hope, identity, meaning and empowerment. Connectedness was a key topic of discussion. Students described they felt a shared understanding, less alone, less like an outsider, increased ability to talk openly, and that the RCB made them feel like being part of a community. The latter they empathised by comparing the RCB to a small village or a social club. Another subtheme mentioned repeatedly across both focus groups was the positive impact of sharing experiences as well as learning from shared experiences.

Yes, and simply this feeling of togetherness, sitting in the same boat and rowing together. (..) Through this I've found a connection to others again and also felt the courage to be able to talk again. (Focus group 2, paragraph 3)

Participants also mentioned changes regarding hopes and motivation after participating in RCB courses. Being thankful for reaching small steps and goals, focusing on positive things in life, and realising that recovery is possible were common themes. There was also a lot of agreement in one of the focus groups that the existence of the RCB resulted in a feeling of hope in general.

The time here at the Recovery College has given me a lot of courage to conquer my path in life and to have confidence (..). (Focus group 1, paragraph 52)

For me, the very existence of the Recovery College is such a hopeful thing, (..) it is such a glimmer of hope for the whole system. (Focus group 2, paragraph 102)

Another theme in both focus groups was the participants finding a more positive attitude towards their own identity again. This involved subthemes such as learning more about oneself, feeling more confident to stand up for wishes and rights, developing a stronger capacity for self‐reflection, and minimising the tendency to adopt a victim mentality. Someone also mentioned realising that their fluctuating self‐esteem is mainly a symptom of their depression and not a part of their identity.

And I now have the feeling, because I have learned several things about myself and learned to deal with myself better, I can also communicate it better to others. (Focus group 1, paragraph 39)

The participants further talked about how the RCB helped them to find new meaning in their lives and to shift some of their negative perspectives. There was common sense among several students that they learned to focus less on their illness, but on things that they enjoy in life. Some explained that they found new purposes in their experiences with mental illness.

You don't always have to know where life is going. But then you say it's nice to live, and (before) I haven't been able to say that for a long time. Now I have the most important things under control. (Focus group 1, paragraph 42)

According to multiple participants, attending RCB courses empowered them to rekindle past hobbies and adopt healthier habits. Subthemes regarding the theme of empowerment included helpful strategies learned in courses, the positive impacts of learning from a person with lived experience, and feeling more in control of one's life. There was also a general appreciation of there not being a right or wrong in RCB courses but a set of opinions, ideas and methods that everyone can adapt to themselves. Moreover, some participants explained that the RCB empowered them by providing a secure environment to practice real‐world scenarios, such as speaking in front of many people.

(..) I have finally managed to be more active in sports and this has been a process over several courses (..) So I feel like they were really encouraging. (Focus group 1, paragraph 22)

### 3.2 Well‐Being

The WHO‐5 sum score increased from 12.4 to 13.7 (difference of 4.8%) demonstrating a significant improvement (*t* (88) = −2.526, *p* = 0.007, *n* = 89) with a low to medium effect size (*r* = −0.268). According to the Levene's test, the variances were homogenous (*p* = 0.616). No statistical difference between the WHO sum score of participants attending more than one course compared to only one was evident after adjusting for the number of courses attended.

(*F* (1, 87) = 0.33, *p* = 0.567, partial *η* ^2^ = 0.04).

Participants from both focus groups gave insights on how the RCB improved their well‐being. Three subthemes were identified: Gaining strength and energy through the courses, feeling better and more positive after finishing a course, and the accomplishment of participating in something like the RCB resulting in improved well‐being. There were also statements on how there will always be better and worse days when struggling with mental illness but that the RCB can be a good place to find support and assurance on these bad days.

And sometimes I came (to a course) when I was feeling like shit, excuse the expression. And I realized that if you can talk about it or go out in that state (..) it can get better. And that really made me feel good (..). (Focus group 2, paragraph 5)

### 3.3 Self‐Stigmatisation

The sum score of the SSMIS‐SF decreased from 13.7 to 11.9 (difference of 4.4%). A significant improvement in these scores was shown in the Wilcoxon analysis (*z* = −3.758, *p* < 0.001, *n* = 90) with a low to medium effect size (*r* = 0.269). Levene's test showed that the variances were not homogenous (*p* = 0.022); therefore, the ANCOVA analysis was not conducted for the SSMIS‐SF.

During the discussion, participants reported that the RCB helped them with one of the main challenges in improving self‐stigmatisation: Recognising when it happens and learning to reflect on it. They emphasised that this was an important first step, although many of them had been struggling with self‐stigmatisation for years, making change difficult. Recurring subtopics were identifying negative thought patterns, working on expectations towards oneself, learning to use positive affirmations instead of negative ones, and acknowledging that small steps can lead to significant progress over time. Co‐production was also mentioned regarding self‐stigma: Students reported feeling seen as equals by course moderators and encountering moderators with lived experience that became role models because of how they turned their experiences with mental illness into something positive.

For me, that was terrific. That was the beginning for me, to be able to recognize self‐stigmatization (..), I'd say, to rethink these self‐destructive thoughts a bit. Of course, you can't just get out of it so easily, I'm still working on it. (Focus group 2, paragraph 45)

Regarding the quantitative evaluation, two students mentioned finding it difficult to measure the impact of the RCB through the pre‐post‐questionnaires. They both stated that several questions mainly reflected a momentary snapshot and that they might felt bad on the day of the evaluation although this had nothing to do with the RCB course or the impact the course had on them. One person then explained this being the reason they wanted to participate in the focus group, to have a chance to elaborate on this.

## 4 Discussion

The results gained through this evaluation indicate that the RCB has several positive impacts on its students' mental health. Significant improvement was found in all three standardised questionnaires, showing improved personal recovery, well‐being and self‐stigmatisation with the complementary qualitative evaluation supporting these results by showing positive impacts across all three themes. Participants reported feeling less alone, a sense of belonging to a community, a more positive attitude towards their life and identity, increased engagement in hobbies and healthy behaviours, increased well‐being and improved ability to recognise and tackle self‐destructive and self‐stigmatising thought patterns and behaviours. A higher number of attended courses did not result in higher levels of the standardised outcomes in the statistical analysis.

Our findings are consistent with the existing literature summarised in the literature review of Thériault et al. (2020). They analysed both qualitative and quantitative studies and found that RCs lead to improved personal recovery and well‐being as well as decreased self‐stigmatisation. Similar results were also found in a recent evaluation study of a Canadian Recovery College (Arbour et al. 2023) showing statistically significant improvement in all three outcome measures, using the QPR to measure personal recovery and two other standardised questionnaires to measure self‐esteem and well‐being.

In addition to statistical significance, it is essential to consider clinical relevance. On average, there was a shift of about 4%–5% towards the desired direction for each questionnaire across students which corresponded to low to medium effect sizes. For the QPR, the recommended minimal difference of 5 points (8.3%) within subjects suggested for clinical relevance was not met in our results, which showed a difference of 2.5 points (4.2%) (Dehmahdi et al. 2021). Similarly, the WHO‐5 showed an improvement of 4.8% in our findings which is lower than the suggested 10% score difference for clinical relevance in this questionnaire (Topp et al. 2015). When considered alongside the low to medium effect sizes observed in the results of all three questionnaires, these findings should be interpreted with caution. Nevertheless, our analysis indicates that approximately one‐third of respondents achieved results within the clinically relevant range, with this figure rising to over 40% for the WHO‐5. This represents a considerable proportion.

Even if the above discussion calls for some caution in the interpretation of the quantitative results, the integrated qualitative results can confirm the quantitative trends that have been shown. The qualitative part of the study provided important insights for a more in‐depth understanding of the quantitative results. They indicate and support the quantitative results that participation in the RCB has resulted in tangible improvements in personal recovery, well‐being and self‐stigmatisation of the participants. The students reported positive impacts in all five CHIME categories, almost exclusively describing beneficial effects that they directly related to their experiences in the RCB. These comments align with previous findings of qualitative evaluations of RCs. Whish, Huckle, and Mason (2022) summarised the qualitative evidence on RCs in their thematic synthesis: Among other they found that students reported a shift in their lives towards increased social inclusion and connection, facilitation of personal growth, increased engagement with their lives, as well as positive outcomes of sharing and learning from experiences of others. Furthermore, our qualitative results confirmed the improvements seen on the self‐stigma questionnaire. They indicate a positive change in the process of reducing self‐stigma while being a student in the RCB, corresponding to the findings of Nurser, Hunt, and Bartlett (2017). Similarly, a Norwegian study showed that RCs may lead students to experience an inner repositioning from having a low self‐worth towards being a person of value and resources (Selbekk et al. 2023). As Whitelaw et al. (2022) have demonstrated, the decreased self‐stigma is likely related to the use of the method of co‐production in RCs, which was also mentioned as an amplifying factor in our focus groups. Moreover, a recent literature review (Woolsey and Mulvale 2022) found that RCs, as interventions outside the formal mental health services, have the potential of attaining increased well‐being as population‐level objectives of policy agendas. Our qualitative and quantitative findings support this by showing that the RCB contributes to students' improved well‐being. Consequently, in the context of Switzerland, policy agendas such as the health policy strategy 2020–2030 of the Federal Council (FOPH 2019) might be influenced positively through the RCBs beneficial effects on well‐being.

It is relevant to consider that the participants' scores on the questionnaires were already quite high at baseline, which likely influenced our findings. In the study of Elhilali et al. (2023), the QPR was answered by a comparable sample including 200 people with mental illness in Switzerland with a similar age and gender distribution. The respondents' mean value of the QPR sum score was 37.2 (SD 9.3) compared to the baseline sum score of the RCB sample with 40 points (SD 8.8). The higher baseline score suggests that people attending the RCB may already have a higher understanding of personal recovery than the average population of people with lived experience before starting a course. The RCB students' psychological well‐being can be viewed in a similar context, as their baseline WHO‐5 scores were more than 7% higher than those of a sample of psychiatric outpatients in Spain with a similar age and gender distribution (Lara‐Cabrera, Mundal, and De Las Cuevas 2020) and only 2% lower than a sample across three countries in the European general population (Carrozzino et al. 2022). For the SSMIS‐SF a comparable sample of Czech psychiatric outpatients had a sum score 7.3% higher than the RCB respondents, suggesting that our sample has a rather low level of self‐stigmatisation even before starting courses (Kalisova et al. 2018). These comparisons may indicate that it is harder to reach clinically relevant changes as there is less room for improvement with high baseline scores. One possible explanation for the high baseline scores could be that students with lower levels of mental health may have been less likely to participate in the evaluation, as it can also be concluded from the qualitative interviews. Even though we did not collect any corresponding data on the participants, our impression during the interviews was that the participants were already well advanced in their recovery process and that the composition was not representative and less diverse in relation to the composition of participants in the courses. Additionally, these results suggest that efforts need to be taken to reach vulnerable groups of people who are not yet familiar with recovery topics, in order to increase their enrollment as potential RCB students.

Analysis comparing scores of the QPR and WHO‐5 across different numbers of courses attended did not show any significant changes, suggesting that a higher number of attended courses does not result in a bigger impact on RCB outcomes. However, given that the majority of participants only attended one or two courses in the RCB, it is difficult to determine whether attending a higher number of courses, such as five or more, would show a different effect. In the qualitative part of the study, half of the participants had attended two or more courses. The very positive results from the interviews could indicate that the number of courses could have a positive influence on the selected outcomes. In a study conducted by Sommer et al. (2019), which assessed a RC based on personal goal achievement, it was observed that attending a greater number of courses had a beneficial effect on students' ability to achieve their goals.

The underlying study has some limitations that need to be acknowledged. First, the selected design of a pre‐post‐evaluation limits the possibilities to control influencing factors in the participant's life. There is a chance that other influences have affected the results in all possible directions, which was also reported by students in the focus groups. Therefore, it is impossible to make a clear statement about the causal effect of the courses on participants' outcomes. Including a control group would have been beneficial; however, this was not possible because of the setting as an evaluation of an existing course programme making it unfeasible to deliver the intervention exclusively to a portion of the participants as well as due to time and financial restraints. A control group selected out of a similar sample with propensity score matching (Austin and Stuart 2015) might have potential for evaluations for the future. Second, students whose mental health declined or who did not make positive experiences in the RCB might have dropped out of courses early and were therefore not included in this study as they won't have completed both questionnaires. This might have led to an overestimation of the impact in our results. Furthermore, we did not collect data on individuals who met the inclusion criteria but did not agree to participate in the study. Providing this number would have offered insights into the extent of potential selection bias. Lastly, although team members with lived experience were involved in the data gathering during the courses, the development of the evaluation of the RCB was not done in co‐production. As Lin et al. (2023) recommend, people with lived experience should be involved in all parts of future evaluations in RCs. A co‐produced evaluation concept could bring to light new ideas on how to evaluate RCs in an appropriate way.

## 5 Conclusion

This is the first standardised evaluation of a RC in Switzerland. Findings show that this form of educational mental health service has the potential to improve the lives of people with mental illness by generating a positive impact on personal recovery, well‐being and self‐stigmatisation. Although the clinical significance of the quantitative findings alone may be debatable, the qualitative findings confirmed that participants experienced concrete improvements because of the RCB in all outcomes measured. This evaluation indicates that RCB students already have a rather high level of knowledge on RC topics at baseline. No implications can be made that a higher number of courses attended leads to a higher effect on outcomes.

## 6 Relevance for Clinical Practice and Research

The positive impact implies that RCs should be made available continuously and further developed also in other regions of Switzerland. Efforts need to be taken to increase outreach to potential students who are not yet familiar with RC topics. Similar projects must be evaluated continuously in this early stage of development to evaluate effectiveness and guarantee quality improvement. To accurately measure the direct impact of the RC on participants, it's essential to use appropriate evaluation designs that may differ from those used in traditional intervention research approaches. These evaluations should be developed and carried out in co‐production. Additionally, in these evaluations impacts on professionals, careers or others should be assessed as they are also students of RCs. Further exploration of the impact of attending multiple RCB courses should be conducted in future evaluations.

## Author Contributions

**Nora Ambord:** data collection and preparation, conducting focus group, analyses, writing manuscript. **Christian Burr:** data collection, conducting focus group, analyses, reviewing manuscript. **Gianfranco Zuaboni:** data collection, reviewing manuscript.

## Conflicts of Interest

The authors declare no conflicts of interest.

## Data Availability

The data that support the findings of this study are available on request from the corresponding author. The data are not publicly available due to privacy or ethical restrictions.
